# Photocatalytic Reduction of Cr (VI) over g-C_3_N_4_ Photocatalysts Synthesized by Different Precursors

**DOI:** 10.3390/molecules26227054

**Published:** 2021-11-22

**Authors:** Juan Liang, Chengjun Jing, Jiarong Wang, Yupawang Men

**Affiliations:** 1 College of Architecture and Environment, Sichuan University, Chengdu 610065, China; ilyq1119@163.com (J.L.); wjrscu@163.com (J.W.); 18988222020@163.com (Y.M.); 2 Institute for Disaster Management and Reconstruction, Sichuan University, Chengdu 610065, China

**Keywords:** Cr (VI) reduction, photocatalytic, g-C_3_N_4_ precursors, nanosheet morphology, surface characteristics

## Abstract

Graphitic carbon nitride (g-C_3_N_4_) photocatalysts were synthesized via a one-step pyrolysis process using melamine, dicyandiamide, thiourea, and urea as precursors. The obtained g-C_3_N_4_ materials exhibited a significantly different performance for the photocatalytic reduction of Cr(VI) under white light irradiation, which is attributed to the altered structure and occupancies surface groups. The urea-derived g-C_3_N_4_ with nanosheet morphology, large specific surface area, and high occupancies of surface amine groups exhibited superior photocatalytic activity. The nanosheet morphology and large surface area facilitated the separation and transmission of charge, while the high occupancies of surface amine groups promoted the formation of hydrogen adsorption atomic centers which were beneficial to Cr(VI) reduction. Moreover, the possible reduction pathway of Cr(VI) to Cr(III) over the urea-derived g-C_3_N_4_ was proposed and the reduction process was mainly initiated by a direct reduction of photogenerated electrons.

## 1. Introduction

In recent years, with the rapid development of mining, electroplating, leather tanneries, and pigments, a large number of heavy metal compounds have been discharged. Among of them, hexavalent chromium Cr(VI) is one of the most virulent contaminants which can be accumulated by digestion system via the exposure and intake the polluted substance, causing serious illness such as cancer and skin allergy [1,2,3]. Because of its high toxicity, Cr(VI) has been ranked among the top 20 toxic pollutants on Superfund Priority List of Hazardous Substances [4]. Photocatalytic reduction technology is an attractive alternative technology for Cr(VI) reduction because of its acceptable cost, easy operation, and high safety. Specifically, it can directly reduce high toxic Cr(VI) to less harmful Cr(III), which is a necessary trace element for human being and easy to precipitation in aqueous solution ($K_{sp}^{\theta }$ (Cr(OH)_3_) = 6.3 × 10^−31^) [5,6,7].

As a metal-free semiconductor photocatalyst, graphitic carbon nitride (g-C_3_N_4_) has been widely studied due to its visible-light-driven, narrow bandgap, non-toxic, low cost, and excellent stability [8,9]. In addition, the conduction band of g-C_3_N_4_ level is much more negative than that of Cr(VI)/Cr(III) (1.3 eV vs. NHE), suggesting that the photo-generated electrons in g-C_3_N_4_ possess a large thermodynamic driving force to reduce Cr(VI) to Cr(III) [10,11]. In general, g-C_3_N_4_ can be synthesized via thermolysis method from cheap available nitrogen-rich precursors such as urea, thiourea, cyanamide, dicyandiamide, and melamine. Due to the different chemical structures of these nitrogen-rich precursors, as well as the influence of foreign chemical elements such as O and S, the g-C_3_N_4_ photocatalysts prepared from these nitrogen-rich precursors exhibit different structural and optical properties, and thus display different photocatalytic activities [12,13,14]. 

Several studies have been conducted to investigate the effect of various precursors on photocatalytic performance and the characteristics of g-C_3_N_4_. However, the field of application of g-C_3_N_4_ derived from diverse precursors is debatable in these studies. Tian et al. found that uran-derived g-C_3_N_4_ exhibits superior photocatalytic activity for tetracycline degradation under visible light when compared with thiourea and dicyandiamide [15]. Tim et al. found thiourea served as the best water-dispersible photocatalyst for MB degradation [16]. Guan et al. founded that g-C_3_N_4_ synthesized from melamine and annealed in N_2_ exhibited much higher catalytic activities for PMS activation rather than urea or thiourea [17]. Furthermore, the correlation between the catalytic activities and the properties g-C_3_N_4_, such as structure, morphology, photochemical, and electrochemical properties is frequently incomplete [18]. Martha et al. found the charge separation property is more relevant than the specific area in the photocatalytic hydrogen evolution [19], while Martin et al. showed the large surface area and porous structure are the main factors for the high hydrogen evolution rate [20]. Ismael et al. claimed that g-C_3_N_4_ with a low polymerization degree has greater photocatalytic activity [21], while Ibad et al. claim that high crystallinity combined with mesoporosity yield highest active g-C_3_N_4_ [22]. Therefore, understanding the impacts of different precursors on the photocatalytic reduction of Cr(VI) and the characteristics of g-C_3_N_4_ will be useful for rationally designing of g-C_3_N_4_ with good photocatalytic performance.

Until now, most of the investigations on photocatalytic reduction of Cr(VI) have focused on the modification of g-C_3_N_4_ material itself, such as copolymerization [23], exfoliation [24], doping [25], heterostructure fabrication [26], etc. There have been few studies on the impact of using various nitrogen-rich precursors. In this paper, g-C_3_N_4_ photocatalysts were synthesized via a thermal polymerization technique with dicyandiamide, melamine, thiourea, and urea as precursors. The obtained products were named as D-CN, M-CN, T-CN, and U-CN, respectively. The resulting catalysts’ structure, morphology, surface properties, photoelectrochemical properties, and the photocatalytic reduction performance of Cr(VI) were further investigated. It was found that urea-derived g-C_3_N_4_(U-CN) exhibits the highest activity due to the nanosheet morphology, large specific surface area, and high occupancies of surface amine groups. Further, these results demonstrate that the specific surface area and surface characteristics play a more predominant role in influencing photocatalytic reduction of Cr(VI) than the photoelectronic properties. Finally, a putative Cr(VI) to Cr(III) reduction pathway in U-CN was hypothesized.

## 2. Results and Discussion

### 2.1. Structure and Morphology

**Figure 1:**
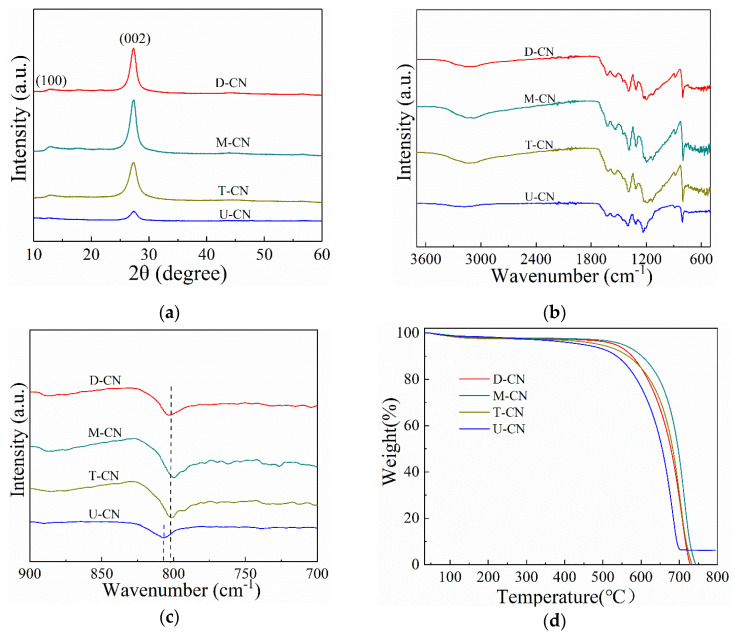
(**a**) XRD patterns; (**b**) FT-IR spectra; (**c**) partial enlarged FT-IR spectra, and (**d**) TG curves of g-C_3_N_4_ prepared by different materials.

Figure 1a shows X-ray diffraction (XRD) patterns of g-C_3_N_4_ prepared by different precursor systems. It can be seen from the XRD pattern that the g-C_3_N_4_ obtained by the four precursors all have two characteristic peaks. The weak peak near 12.8° is attributed to intralayer long-range atomic order (100), which is associated with the hydrogen bonds in g-C_3_N_4_ [27,28]. The stronger peak near 27.4° corresponded to the (002) crystal plane of g-C_3_N_4_, which is caused by the interlayer accumulation of the conjugated aromatic system [29]. These characteristic diffraction peaks are consistent with previous reports. In addition, the diffraction peaks of U-CN are broader and lower intensity, indicating that U-CN has the lowest crystallinity. The low crystallinity of U-CN might be that extra O in urea produces H_2_O, CO_2_, and ammonia during calcination, which inhibit the growth of the surface crystal [30]. Furthermore, the (100) crystal plane of U-CN is substantially lower than the other three samples, indicating a reduced hydrogen bond effect in the intralayer of U-CN [20].

Figure 1b presents the FTIR spectra of the g-C_3_N_4_ samples to demonstrate their graphitic structures. The absorption peaks observed between 1200–1700 cm^−1^ correspond to the characteristic breathing modes of aromatic carbon nitride heterocyclic rings [31]. The sharp absorption band at around 801 cm^−1^ is attributed to the respiratory pattern of triazine units while the board vibration bands at 3000–3500 cm^−1^ can be ascribed to the uncondensed amine groups and the water molecules adsorbed on the surface [14]. In addition, it can be seen from Figure 1c that U-CN blue-shifted at 801 cm^−1^. The main reason might be due to the hydrogen bond-containing which influences the triazine ring stretching in the g-C_3_N_4_ structure [16].

The lower degree of polymerization of U-CN can also be obtained from thermal stability of the different as-prepared g-C_3_N_4_. It can be seen from Figure 1d that there is no further significant weight loss up to a temperature of 400 °C. However, weight losses of 21.1%, 29.0%, 33.3%, and 53.5% were observed for the M-CN, T-CN, D-CN, and U-CN between 400 °C to 650 °C, respectively. U-CN can be completely decomposed at 700 °C, while the completely decomposition temperature of T-CN, D-CN, and M-CN are 728 °C, 732 °C, and 746 °C, respectively. The result suggest that U-CN has the worst thermally stability, which might be due to its low degree of polymerization and poor stability of the triazine ring structure.

**Figure 2:**
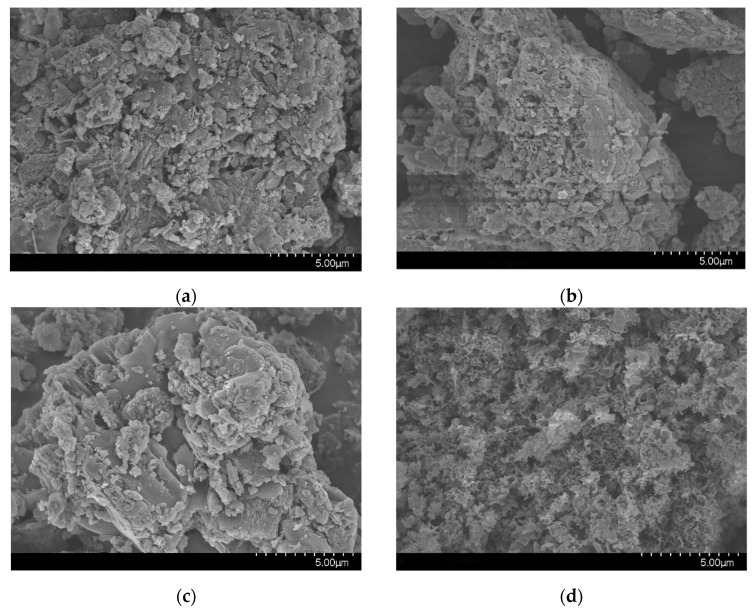
SEM of g-C_3_N_4_: (**a**) D-CN, (**b**) M-CN, (**c**) T-CN, and (**d**) U-CN.

The morphologies of the prepared samples were investigated by SEM. D-CN and M-CN show the typical flat and layered structure with small lamellas wrapped in large particles (Figure 2a,b), whereas T-CN has an obvious layered structure, with large lamellae and a few fine particles scattered on the surface (Figure 2c). U-CN displays nanosheets morphology with irregular wrinkles (Figure 2d) [32]. It can also be observed that U-CN displays porous structure while the last three sample present large sheet without porous structures. 

**Figure 3:**
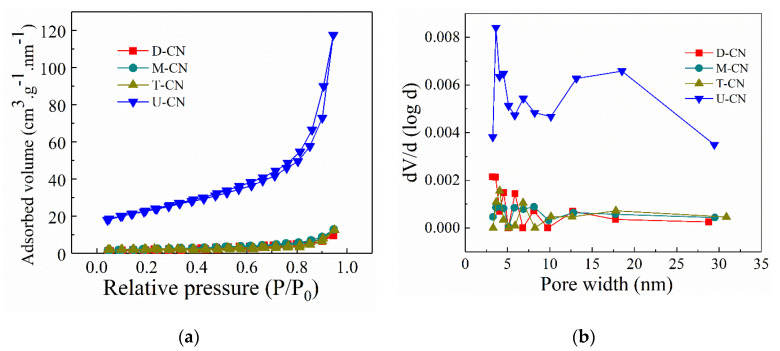
(**a**) N_2_ adsorption-desorption isotherms and (**b**) corresponding pore size distribution curves of D-CN, M-CN, T-CN, U-CN.

The surface area and porous structure of the prepared samples were further studied on the basis of nitrogen gas adsorption–desorption isotherms and pore size distribution curves. As shown in Figure 3, all the samples exhibit a classical type IV isotherms, which is the characteristic of the typical mesoporous materials. The BET surface areas and pore volume of the as-prepared samples were summarized in Table 1. It can be seen that U-g-C_3_N_4_ has the largest specific surface area (S_BET_ = 81.060 m^2^ g^−1^) and pore volume (V_meso_ = 0.164 cc g^−1^), while the BET surface areas and pore volume of D-CN (S_BET_ = 8.779 m^2^ g^−1^, V_meso_ = 0.025 cc g^−1^), M-CN (S_BET_ = 8.363 m^2^ g^−1^, V_meso_ = 0.021 cc g^−1^), and T-CN (S_BET_ = 7.262 m^2^ g^−1^, V_meso_ = 0.019 cc g^−1^) have little difference. These results are in good agreement with the morphologies of the samples. The presence of the oxygen heteroatom in urea might also play an important role in increasing the BET surface area and pore volume of the U-CN sample. The emission of pyrolysis-generated gases during the thermal condensation process can function as soft templates and promote the formation of porous structure [33,34]. The external surface area is very important in photocatalytic action as the reactions take place mainly on the external surface that is exposed to light irradiation.

**Figure 4:**
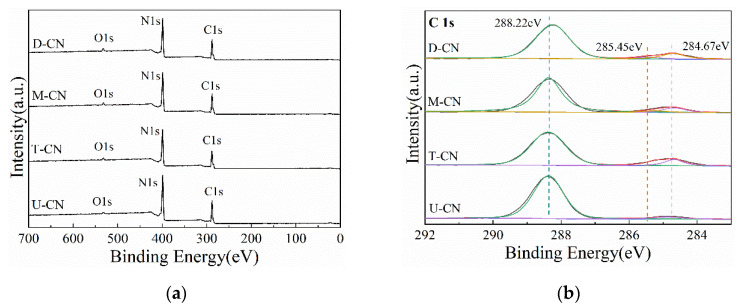

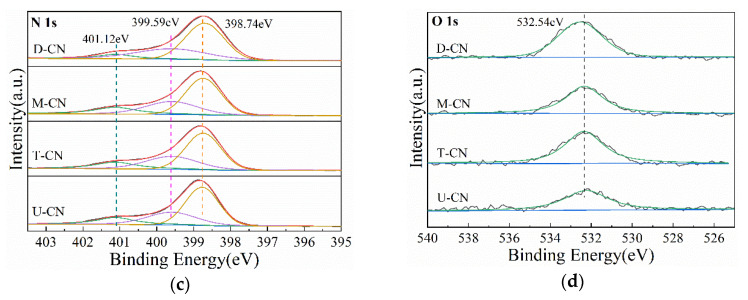
XPS spectra of the samples: (**a**) survey, (**b**) C1s, (**c**) N1s, and (**d**) O1s.

The XPS spectra of the samples are presented in Figure 4. The survey scan XPS spectra shown in Figure 4a illustrates that the obtained g-C_3_N_4_ samples are composed of C, N, and O elements. It can also be seen from Figure 4 that C1s, N1s, and O1s have no obvious energy shifts while core electrons occur, indicating the chemical states of the three elements are the same in D-CN, M-CN, T-CN, and U-CN. As shown in Figure 4b, the high-resolution XPS spectra of the C 1s for the samples can be deconvoluted into three peaks with binding energies of 284.67 eV, 285.45 eV, and 288.22 eV, which are characteristic of the sp^2^ C-C bond, C-O bond, and N-C=N bond, respectively [35]. The high-definition N 1s spectra of the samples can be fitted into three distinct peaks at 398.74 eV, 399.59 eV, and 401.12 eV, corresponding to C-N=C, tertiary nitrogen N-(C)_3_ and -NH_2_ (Figure 4c) [36]. In addition, a weak energy peak at 532.54 eV can also be observed in the high resolution XPS spectra of O1s, which can be attributed to adsorbed H_2_O on the sample surface [37] (Figure 4d). 

The surface elements content of the samples can be analyzed from the integrated peak areas under C1s, N 1s, and O 1s (Table 1). The atomic ratios of C/N of D-CN, M-CN, T-CN and UCN are determined to be 0.82, 0.81, 0.86, and 0.72, respectively. The C/N ratio of U-CN is the lowest, implying a more defective structure and lower polymerization. The zeta potentials of the samples were measured to investigate the surface charges of the samples. In the suspension with the initial pH, the zeta potentials of D-CN, MCN, TCN, and U-CN are −21.45 eV, −21.75 eV, −23.31 eV, and −15.13 eV, respectively. g-C_3_N_4_ contains abundant Lewis acid and base sites, which are derived from the terminal and bridging NH-groups and lone pairs of N in triazine/heptazine rings, respectively. Amine groups can act as proton acceptors and acquire positive surface charges. Additionally, hydroxyl ions can react with primary and secondary amine groups to produce negative charges on the surface of g-C_3_N_4_. The surface charges and zeta potentials of g-C_3_N_4_ are determined by the number of amine groups on the carbon surface and the pH value of the suspension [38]. The amine groups on the U-CN surface are substantially higher than those on the D-CN, M-CN, and T-CN because of the huge specific surface area and much higher surface N element concentration, resulting in a much stronger ability to adsorb hydrogen ions in the solution with the same pH value. Furthermore, Lewis acid and base sites on the surface of g-C_3_N_4_ are potential anchoring sites for cocatalysts [39]. A higher occupation of surface amine group may provide more active sites for the photocatalytic reaction.

### 2.2. Photocatalytic Performance

**Figure 5:**
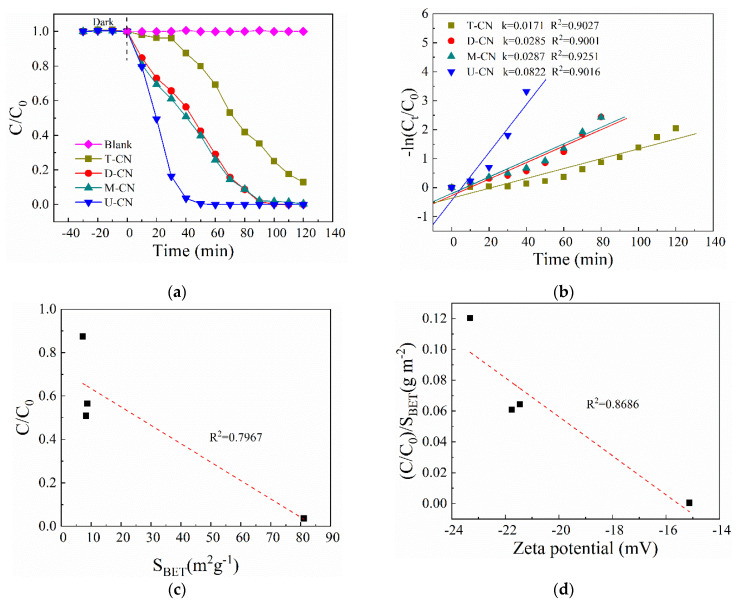
(**a**) Effect of materials on the photocatalytic activity of g-C_3_N_4_, (**b**) Photodegradation kinetic constants of g-C_3_N_4_ prepared from different materials, (**c**) Correlation of C/C_0_ and specific surface area (S_BET_), (**d**) Correlation of N content and normalized C/C_0_ (k/S_BET_). (Experimental conditions: initial Cr(VI) concentration = 50 mg L^−1^; catalyst amount = 50 mg; reaction volume = 150 mL; citric acid = 0.9 Mm; pH = 3; white light irrigation).

The photocatalytic reduction performance of Cr(VI) over the as-prepared photocatalysts were evaluated under white light irradiation. As shown in Figure 5a, due to the weak electrostatic attraction between the anionic chromate species (HCrO_4_^−^ and/or Cr_2_O_7_^2−^) and the negative charge on the surface of the g-C_3_N_4_ catalysts, all the samples show poor adsorption capacity for Cr(VI). Under the irradiation of white light, all the samples display significantly different photocatalytic performance, in which U-CN exhibits the best Cr(VI) reduction activity with efficiency of 99.5% within 60 min, while the efficiencies are 71.1%, 74.1%, and 30.7% for D-CN, M-CN, and T-CN, respectively. Figure 5b depicts the kinetic curves of the photocatalytic reduction of Cr(VI) with the typical pseudo-first-order model (lnC/C_0_ = −kt). The rate constants of U-CN, D-CN, M-CN, and T-CN are 0.0822 min^−1^, 0.0285 min^−1^, 0.0287 min^−1^, and 0.0171 min^−1^, respectively. Obviously, U-CN possesses the highest rate constant, which corresponds to the best photocatalytic performance.

Combined with the previous morphological structure and surface characteristics analyses, it was found that the photocatalytic reduction effect of the catalyst has a certain correspondence with its specific surface area. As shown in Figure 5c, a close correlation was found between the C/C_0_ and S_BET_ with a correlation coefficient 0.7967. U-CN with nanosheet morphology, large specific surface area has the best photocatalytic reduction performance. The large surface area facilitates the separation and transfer of photoinduced charges in U-CN, as well as provide more active sites for Cr(VI) reduction. In addition, it is worth noting that, despite having a similar surface area as D-CN and M-CN, T-CN had the lowest photocatalytic reduction activity. By analyzing the surface characteristics of the three samples, it is found that the surface amine group content of the samples has a significant impact on the photocatalytic activity. The surface amine groups can act as exciton dissociation traps which are conducive to the rapid splitting of photogenerated excitons and promote the formation of hydrogen adsorption atomic centers, thus facilitating the photocatalytic reduction of Cr(VI) [39]. The effect of S_BET_ is excluded while investigating the possible role of surface amino groups in the photocatalytic reduction process. Figure 4d shows the association between normalized C/C_0_ ((C/C_0_)/S_BET_) and zeta potential. As illustrated in Figure 4d, the value of normalized C/C_0_ is closely correlation with the zeta potential which is determined by the varied specific area and the occupation of the surface amine groups [38]. The correlation coefficient is conculcated to be 0.8686. The results reveal that the photocatalytic reduction activity of Cr(VI) is affected by the specific surface area and the amount of surface amine groups, and the amount of surface amine groups has a greater impact.

**Figure 6:**
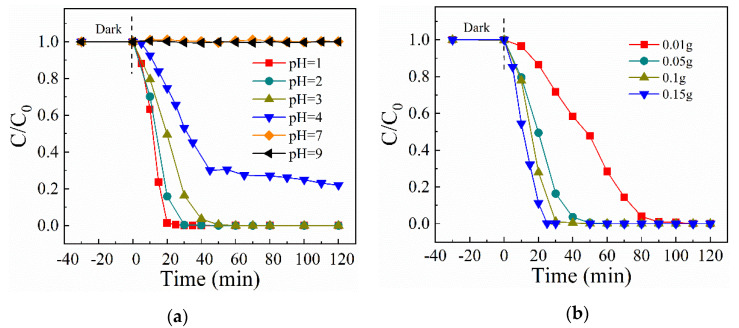
Effect of (**a**) initial pH and (**b**) U—CN dosage on photocatalytic Cr(VI) reduction (Vary conditions are based on the control experiment: Cr(VI) = 50 mg/L; U—CN mount = 50 mg; reaction volume = 150 mL; citric acid = 0.9 Mm; pH = 3; white light irrigation).

Figure 6a depicts the effect of initial pH of the solution on Cr(VI) reduction over U-CN. The photocatalytic reduction efficiency of Cr(VI) decreases markedly with the increase of pH value. In addition, when pH is 7 or 9, Cr(VI) cannot be degraded at all. pH value affects the existence of Cr(VI) as well as the surface charge of g-C_3_N_4_. Cr(VI) is presented as Cr_2_O_7_^2−^, HCrO_4_^−^ in acidic solution and mainly as CrO_4_^2−^ in basic solution [40]. The surface of the catalyst becomes highly protonated at low pH value, which makes the surface of the catalytic more conducive to the accumulation of HCrO_4_^−^. While the surface of g-C_3_N_4_ is negatively charged at alkaline solution, which tends to repel the Cr_2_O_7_^2−^. The Cr (VI) photoreduction was achieved following Equations (1) and (2) under acidic solution, and the hydrogen ion was beneficial to the reduction reaction. In contrast, the Cr(VI) reduction under alkaline solution was accomplished following Equation (3) [41]. In addition, Cr(OH)_3_ may be formed under high pH value and covers the active sites of the photocatalyst, leading to the declining Cr(VI) reduction performance [42].

(1) $${\mathrm{Cr}}_{2}O_{7}^{2-}+14H^{+}+6e^{-}\;\to 2{\mathrm{Cr}}^{3+}+7H_{2}O$$

(2) $${\mathrm{HCrO}}_{4}^{-}+7H^{+}+3e^{-}\;\to {\mathrm{Cr}}^{3+}+4H_{2}O$$

(3) $${\mathrm{CrO}}_{4}^{2-}+4H_{2}O+3e^{-}\;\to \mathrm{Cr}{\left(\mathrm{OH}\right)}_{3}+5{\mathrm{OH}}^{-}$$

The effect of U-CN dosage was also tested. As shown in Figure 6b, the photocatalytic reduction rate of Cr(VI) increases dramatically as the mount of U-CN catalyst is increased. The improvement in the photocatalytic degradation rate of Cr(VI) declined as the catalyst dosage was increased further. The reason might be that the effect of photocatalytic reaction is related to the catalyst surface’s reaction sites [26]. The catalyst’s reaction sites can be effectively increased by increasing the catalyst dosage. While the surface active sites meet the need for Cr(VI) reduction as the dose is increased, the influence of the catalyst dosage on the total reaction rate of Cr(VI) reduction is lowered.

Furthermore, the Influencing factors on Cr(VI) photocatalytic reduction including light sources, hole scavengers, initial Cr(VI) concentration are presented in Supplementary Material (Figures S2–S4).

### 2.3. Photoelectrochemical Properties

**Figure 7:**
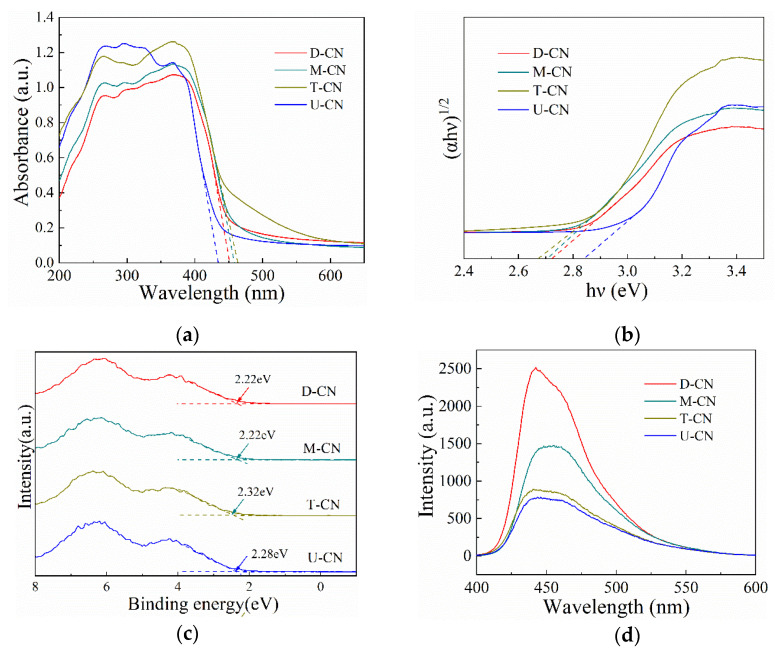
(**a**)UV–vis DRS spectra; (**b**)Tauc plots for estimating the bandgap (Eg) values (**c**) XPS valance band (VB) spectra and (**d**) band structure of D—CN, M—CN, T—CN and U—CN.

Figure 7a shows the UV-visible absorption spectra of the samples. T-CN exhibits stronger light absorption in the range of 400 to 700 nm than that of the other three samples. While the absorption edge of U-CN showed a significant blue shift with respect to the other three samples [16,21]. The band-gap energies of the samples are calculated by plots of (αhv)1/2 versus photo energy. As depicted in Figure 7b, the bandgaps of T-CN, M-CN, D-CN and U-CN are estimated to be 2.68, 2.70, 2.72 and 2.84 eV, correspondingly. U-CN has a larger band gap might be attributed to the quantum size effect caused by smaller and disordered crystalline domains [33,34]. The band structures of the samples were further characterized by valence band XPS(VB-XPS). As shown in Figure 7c, the VB maxima of D-CN, M-CN, U-CN and T-CN are 2.22, 2.22, 2.28 and 2.32 eV, respectively [43]. According to the results of UV-DRS spectra and CB XPS, the VB potential of D-CN, M-CN, T-CN and U-CN are conculcated to be −0.50, −0.48, −0.56, −0.36 eV, respectively. Due to the most negative CB potential in UCN, the photogenerated electrons produced by U-CN have the most reducing ability for Cr(VI) reduction in comparison to those in the other three samples.

**Figure 8:**
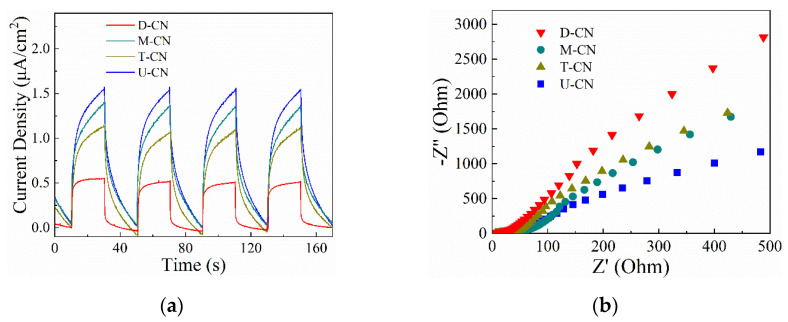
(**a**) Photocurrent response; (**b**) EIS Nyquist plots of D—CN, M—CN, T—CN, U—CN.

The transfer behavior and separation efficiency of photogenerated charge carriers in the samples can be reflected by the photoluminescence (PL) spectra. As shown in Figure 7d, the emission peak centers of the four samples are around 440 nm, which represented the irradiative recombination of e^−^ and h^+^ [21]. The emission intensity is lower for the U-CN as compared to the other three samples, indicating the recombination rate of electrons and holes under white light irradiation is lower in U-CN. To further investigate the separation efficiency of photogenerated charges during the photoreactions, photoelectrochemical measurements were performed. As displayed in Figure 8a, fast photocurrent responses via on-off cycles were observed for all the samples, while the photocurrent intensity of U-CN was obviously higher than that of the other three samples. In addition, the arc radium of U-CN in the EIS Nyquist plot shown in Figure 8b was also smaller than that of the other three samples. It was clear to see that the diameter of arc radius followed in the order of D-CN < M-CN < T-CN < U-CN, which agrees well with the PL spectra.

As mentioned above, the photoelectric properties have little effect on the photocatalytic reduction of Cr(VI). Although having the widest bandgap, U-CN displays the best photocatalytic reduction performance. T-CN has the highest white light absorption and much higher charge separation efficiency, but it displays the lowest photocatalytic reduction activity.

### 2.4. Possible Reaction Mechanism

**Figure 9:**
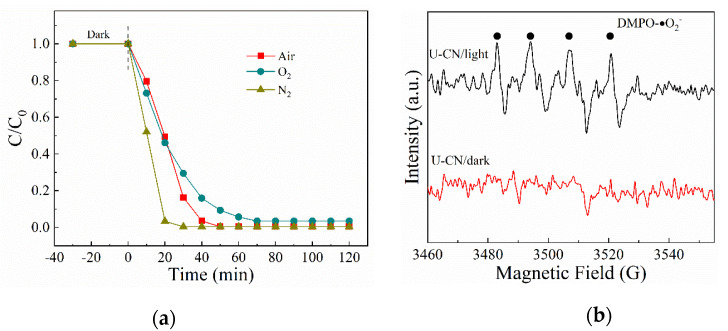
(**a**) Photocatalytic reduction of Cr(VI) in air and N_2_ ambient; (**b**) ESR spectrum of •O_2_^−^.

**Figure 10:**
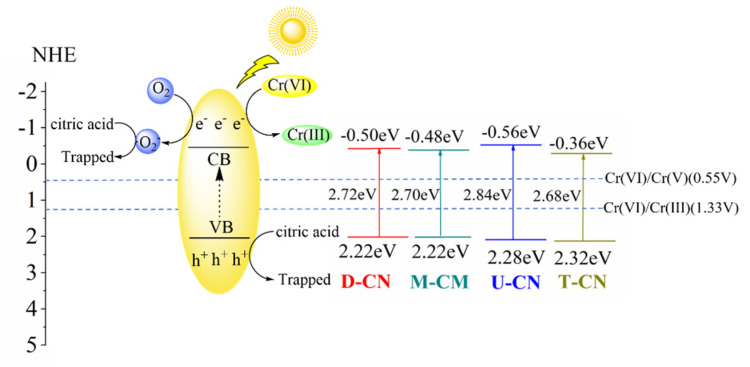
Proposed mechanism for photocatalytic reduction of Cr(VI) over U-CN.

Previous studies have shown that the photocatalytic reduction of Cr (VI) on the surface of g-C_3_N_4_ occurs via a direct or indirect reduction of photogenerated electrons. The trapping of photogenerated electrons by O_2_ is critical for Cr (VI) reduction in the reduction process [3,29,44,45]. To investigate the role of O_2_ on the photocatalytic reduction of Cr (VI) over U-CN in the presence of citric acid, the comparison experiments were carried out in different gas atmospheres. It is found from Figure 9a that the photocatalytic reduction of Cr(VI) over U-CN in the N_2_ atmosphere was obviously enhanced, whereas the photocatalytic reduction of Cr(VI) over U-CN in O_2_ atmosphere was depressed to some extent. The results indicate that O_2_ is involved in the photocatalytic reduction of Cr (VI) over U-CN. ESR technique was further employed to measure the reactive species generated during photocatalysis. As shown in Figure 9b, signals of DMPO-•O_2_^−^ could be detected in methanolic suspension of U-CN under white light irradiation, reflecting that •O_2_^−^ is generated via electron transfer from conduction band of U-CN to the dissolved molecular oxygen under white light illumination. Thus, it can be concluded that in the photocatalytic reduction system, Cr (VI) is direct reduced by photogenerated electrons over U-CN, whereas O_2_ in the solution competes with Cr(VI) for the photogenerated electrons, inhibiting Cr(VI) reduction. While citric acid acts as sacrificial agent of reactive oxygen species in the system, avoiding the Cr(III) re-oxidation, while the surplus electrons will participate in the reaction of Cr(VI) reduction or trapped by O_2_ to form •O_2_^−^ (Figure 10) [46,47].

## 3. Materials and Methods

### 3.1. Materials and Instruments

Melamine, dicyandiamide, thiourea, urea, potassium dichromate(K_2_Cr_2_O_7_), acetone, sulfuric acid(H_2_SO_4_), phosphoric acid(H_3_PO_4_), diphenyl carbonyl hydrazine, sodium hydroxide(NaOH), methyl alcohol, oxalic acid, formic acid, citric acid, and tartaric acid were all analytical grad and obtained from Chron Chemical Reagent Co., Ltd. (Chengdu, China.) Furthermore, 5,5-dimethyl-1-pyrroline N-oxide(DMPO) was supplied by the Aladdin Industrial Corporation (Shanghai, China). All the reagents were analytically pure and the solutions were prepared using ultrapure water.

### 3.2. Preparation of Graphitic Carbon Nitride (g-C_3_N_4_)

The g-C_3_N_4_ samples were synthesized by a thermal polymerization method using dicyandiamide, melamine, thiourea, and urea as precursors. Typically, 10 g of the precursor powder was put into an alumina crucible and heated at 5 °C/min up to 550 °C for 3 h in a covered muffle furnace. After the crucible cooling to room temperature, four products were collected and ground into powder. The obtained products were named as D-CN, M-CN, T-CN, and U-CN, respectively (Figure S1, Supplementary Materials).

### 3.3. Photocatalytic Experiments

The photocatalytic activity of the synthesized samples for the removal of Cr(VI) was evaluated under white light provided by a Xe light with the power of 300 W (Perfectlight). At room temperature a certain amount of g-C_3_N_4_ was suspended into 150 mL aqueous solution of Cr(VI) (50 mg/L) with the addition of citric acid (0.9 mM). The solution pH was adjusted to 3 by H_2_SO_4_ (1 M) or NaOH (1 M). Prior to irradiation, the suspension was ultrasonicated for 10 min and stirred in the dark for 30 min to establish the adsorption–desorption equilibrium. Subsequently, the light source was switched on. During the illumination process, a certain amount of the solution was taken at a predetermined time interval and filtered through a 0.45 μm filter. The Cr(VI) concentration in the supernatant was determined at 540 nm using the diphenylcarbazide (DPC) method by a spectrophotometer at the characteristic wavelength of 540 nm.

### 3.4. Characterization

X-ray diffraction (XRD) patterns were examined using X-ray diffractometer (Panalytical X’Pert-pro MPD, Almelo, The Netherlands) with Cu Kα radiation source (λ = 1.54056 Å) in the range of 10°–60°. The Fourier transform infrared spectra were measured using an infrared spectrometer (Nicolet iS 10, Madison, WI, USA) with KBr pallets. The morphology of the sample was studied using a scanning electron microscope (Hitachi S-4700, Tokyo, Japan). Thermogravimetry (TG) of the samples was analyzed by TA synchronous thermal analyzer ( TA Q600, New Castle, USA). The surface area was calculated using the multi-point BET (Quantachrome NOVA 2000e, Boynton Beach, USA) method. X-ray photoelectron spectroscopy (XPS) was performed using an X-ray photoelectron (ThermFischer ESCALAB 250Xi, Waltham, MA, USA) spectrometer with Al monochrome (hv = 1486.6 Ev) as the X-ray source. The zeta potential of the prepared sample was measured at 293 K using a zeta potential analyzer (Malvern, Zetasizer Nano ZS90, Worcestershire, UK). The UV–vis diffuse reflectance spectra (UV-DRS) were measured on a UV–vis spectrometer (Shimadzu UV-2600, Kyoto, Japan). The photoluminescence (PL) spectra of the samples were recorded by using a fluorescence spectrophotometer (Hitachi F-7100, Tokyo, Japan). The ESR spectra were recorded on a ESR spectrometer (Bruker EMX plus X-band CW, Rheinstetten, Germany) using DMPO as a spin trap agent at room temperature.

The photoelectrochemical measurements were applied in a standard three-electrode cell with an electrochemical workstation (CHI770E, CHN). A Pt and Ag/AgCl (saturated KCl) electrode were chosen as counter and reference electrode, respectively. ITO coated with the prepared catalyst served as the working electrode. The working electrodes were prepared as follows: 5 mg of the as-obtained photocatalyst was suspended in a mixed solution (10 μL of 5% nafion and 2 mL of ethanol) with ultrasound. After that, 100 μL of the obtained suspension was drop-coated on the ITO glass (10 mm × 10 mm), and dried in the air to completely eliminate water. Additionally, 0.1 M Na_2_SO_4_ was used as electrolyte.

## 4. Conclusions

In this study, g-C_3_N_4_ composites are synthesized via a facile polymerized method with four different precursors (i.e., melamine, dicyandiamide, thiourea, and urea). It was found that the type of precursors has a significant impact on the morphology and structure of g-C_3_N_4_ and further affects the performance of photocatalytic reduction of Cr(VI). Urea-derived U-CN with nanosheet morphology, large specific surface area, and high occupancies of surface amine groups exhibit superior photocatalytic activity. These results demonstrate that large surface area and high surface amine groups can provide more catalytically active sites. This work confirms the effect of surface properties on the photocatalytic activity of g-C_3_N_4_ and provides a theoretical and technical foundation for the construction of practical and high-efficiency photocatalysts based on g-C_3_N_4_.

## Figures and Tables

**Table 1:** Specific surface area and pore volume of as-prepared samples.

Name	BET (m^2^ g^−1^)	Pore Volume (cc g^−1^)	Elemental Composition (wt%)				Zeta Potential (mV)	E_gap_ (eV)	k (min^−1^)
			C	N	O	C/N			
D-CN	8.779	0.025	43.85	53.12	2.86	0.82	−21.45	2.72	0.0285
M-CN	8.363	0.021	43.82	53.94	2.24	0.81	−21.75	2.70	0.0287
T-CN	7.262	0.019	45.13	52.19	2.63	0.86	−23.31	2.68	0.0171
U-CN	81.060	0.164	41.58	56.98	1.44	0.72	−15.13	2.84	0.0822

## Data Availability

Data are contained within the article.

## Supplementary Materials

The following are available online. Figure S1: g-C_3_N_4_ photocatalysts prepared by different precursors, Figure S2: (**a**) Photocatalytic removal of Cr(VI) over g-C_3_N_4_ photocatalysts prepared by different precursors (**b**) Photodegradation kinetic constants of the as-prepared photocatalysts under white light and visible light, Figure S3: Effect of hole scavengers on photocatalytic Cr(VI) reduction, Figure S4: Effect of Cr(VI) initial concentration on photocatalytic Cr(VI) reduction.

## Sample Availability

The samples are available from the authors.
