# Supplementary material for: Photocatalytic Reduction of Cr (VI) over g-C3N4 Photocatalysts Synthesized by Different Precursors
Source: Molecules. 2021 Nov 22;26(22):7054. doi: 10.3390/molecules26227054 (PMC8620835; doi:10.3390/molecules26227054)
Supplement: Supplementary file 1 [file molecules-26-07054-s001.zip › molecules-1462141-supplementary.pdf]

molecules

MDPI

Supplementary Materials

Photocatalytic Reduction of Cr (VI) over g-C_3N_4 Photocatalysts Synthesized by Different Precursors

Juan Liang [1,2], Chengjun Jing [1,2,*], Jiarong Wang [1] and Yupawang Men [1]

[1] College of Architecture and Environment, Sichuan University, Chengdu 610065, China; ilyq1119@163.com (J.L.); wjrscu@163.com (J.W.); 18988222020@163.com (Y.M.)
[2] Institute for Disaster Management and Reconstruction, Sichuan University, Chengdu 610065, China
* Correspondence: chengjunjing@126.com; Tel.: +86-18980892979

Cr(VI) measurement

Cr(VI) concentration was determined at 540 nm using the diphenylcarbazide (DPC) method by a spectrophotometer. 4.0 mL of reaction solution was filtered through a 0.45 μm filter. Then the solution was mixed with 0.5 mL of 9.2 mol L-1 H_2SO_4 and 0.5 mL of 7.5 mol L-1 H_3PO_4 and in a 50.0 mL volumetric flask. Subsequently, 2.0 mL of freshly prepared 0.25% (weight/volume) diphenyl carbonyl hydrazine in acetone was added to the volumetric flask, diluting to 50 mL. After the mixture was shaken for about 30 seconds, it was allowed to stand for 10 min to ensure full color development. The absorbance of the colored Cr(VI)-diphenyl carbonyl hydrazine complex solution was measured by a UV-vis spectrometer (Mapada UV-1800) at 540 nm. Cr(VI) concentration under different illumination time was calculated by Cr(VI) standard curve. The photocatalytic reduction efficiency of Cr(Ⅵ) was calculated by the ratio of the concentration C_t obtained at different times to the initial concentration C_0.

EPR measurement

The $\bullet O_2^-$ species were trapped by the DMPO in methanol at room temperature. In detail, 2 mg of U-CN sample was dissolved in 2 mL of methanol ($\bullet O_2^-$), and then 40 μL of DMPO was added. After irradiated under white light for about 5 min, the EPR spectra were recorded on a Bruker EMX plus X-band CW EPR spectrometer.

g-C_3N_4 photocatalysts prepared by different precursors

The g-C_3N_4 samples were synthesized by a thermal polymerization method using dicyandiamide, melamine, thiourea and urea as precursors. The obtained products were named as D-CN, M-CN, T-CN and U-CN, respectively **(FigS1)** .

Effect of light sources on photoreduction of Cr(VI)

Fig. S2a listed the photocatalytic reduction performance of Cr(VI) over g-C_3N_4 photocatalysts synthesized by different precursors under visible light irradiation. Under visible light, U-CN still has the highest photocatalytic activity, followed by D-CN and M-CN, and T-CN has the lowest activity. When the photocatalytic activity of the four samples is compared under visible light and white light, it is clear that the photocatalytic activity of the four samples on Cr has dropped dramatically under visible light(Fig. S1b). This is primarily due to variations in light intensity and wavelength range.

Effect of hole scavengers on photoreduction of Cr(VI)

The role of the addition hole scavengers on the photocatalytic reduction performance was also investigated. As shown in Fig.S3, it is easily seen that the rate of Cr(VI) reduction has the order of Methyl Alcohol (MA) < Oxalic Acid (Ox) < Formic Acid (FA) < Citric Acid (Cit) < Tartaric Acid (TA). Of the tested acids and alcohols, TA is the most efficient, but MA cannot accelerate the photocatalytic reduction of Cr(VI) over g-C3N4. The removal efficiency of Cr(VI) was related with the coordination and electron donating ability of organic sacrificial agents which was similar to the previous reports[34].

Effect of initial Cr(VI) concentration on photoreduction of Cr(VI)

Fig.S4 illustrates the effect of initial Cr(VI) concentration ringing from 10mg/L to 70mg/L on the photocatalytic reduction of Cr(VI) over U-CN at pH 3.0. It can be seen from Fig 10a that with the increase of initial concentration of Cr(VI), Cr(VI) reduction rate and reduction degree is reduced. When the initial concentration of Cr(VI) for 10 mg/L, after 10 minutes it can be fully restored. And when the initial concentration of Cr(VI) for 70 mg/L, respond after 80 minutes (VI) will be restored. The results demonstrated that low initial concentration is beneficial to the photocatalytic reaction, and the catalytic reaction can be accelerated with the reduction of pollutant concentration.

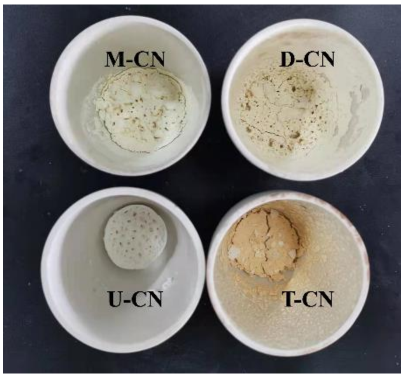

Figure S1 g-C_3N_4 photocatalysts prepared by different precursors

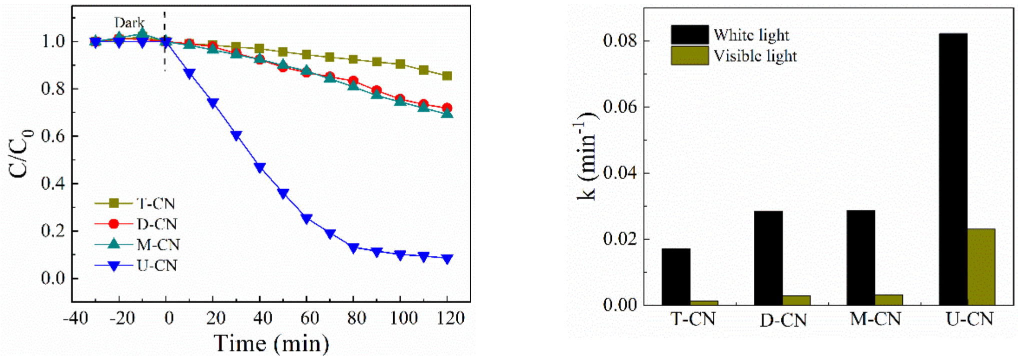

(**a**) (**b**)

Figure S2 (**a**) Photocatalytic removal of Cr(VI) over g-C_3N_4 photocatalysts prepared by different precursors (**b**) Photodegradation ki-netic constants of the as-prepared photocatalysts under white light and visible light(Experimental conditions: initial Cr(VI) concen-tration=50mg L^{-1}; catalyst amount=50mg; reaction volume=150ml; citric acid=0.9Mm; pH=3).

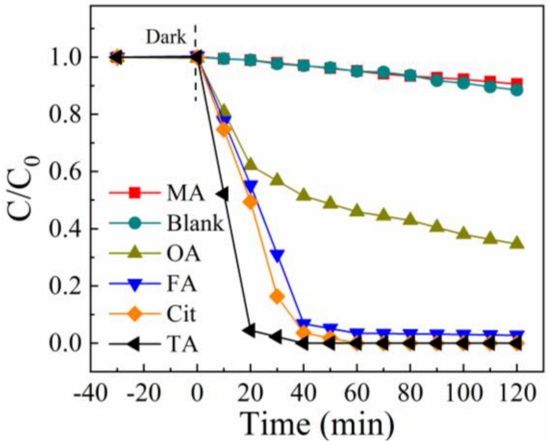

Figure S3 Effect of hole scavengers on photocatalytic Cr(VI) reduction (Vary conditions are based on the control experiment: Cr(VI)=50 mg/l; U-CN mount=50mg; reaction volume=150ml; citric acid=0.9Mm; pH=3; white light irrigation).

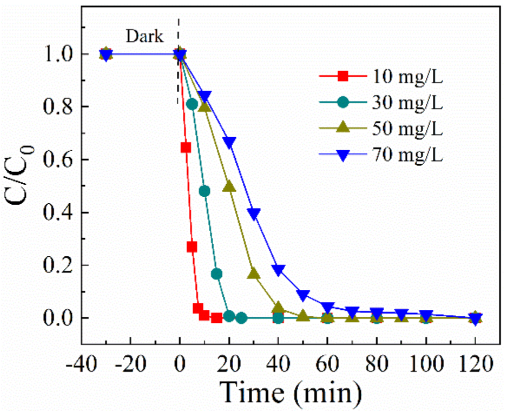

Figure S4 Effect of Cr(VI) initial concentration on photocatalytic Cr(VI) reduction (Vary conditions are based on the control experiment: U-CN mount=50mg; reaction volume=150ml; citric acid=0.9Mm; pH=3; white light irrigation).
